# Role of Endogenous Galectin-3 on Cell Biology of Immortalized Retinal Pigment Epithelial Cells In Vitro †

**DOI:** 10.3390/ijms26157622

**Published:** 2025-08-06

**Authors:** Caspar Liesenhoff, Marlene Hillenmayer, Caroline Havertz, Arie Geerlof, Daniela Hartmann, Siegfried G. Priglinger, Claudia S. Priglinger, Andreas Ohlmann

**Affiliations:** 1 Department of Ophthalmology, University Hospital, Ludwig-Maximilians-University Munich, 80336 Munich, Germany; caspar.liesenhoff@med.uni-muenchen.de (C.L.); m.hillenmayer@campus.lmu.de (M.H.); caroline.havertz@med.uni-muenchen.de (C.H.); siegfried.priglinger@med.uni-muenchen.de (S.G.P.); claudia.priglinger@med.uni-muenchen.de (C.S.P.); 2 Protein Expression and Purification Facility, Institute of Structural Biology, Helmholtz Center Munich for Environmental Health, 85764 Neuherberg, Germany; arie.geerlof@helmholtz-muenchen.de; 3 Department of Dermatology and Allergy, LMU University Hospital, Ludwig-Maximilians-University Munich, 80337 Munich, Germany; daniela.hartmann@med.uni-muenchen.de; 4 Department of Dermatology and Allergy, Munich Municipal Hospital, 80337 Munich, Germany

**Keywords:** galectin-3, galectin-3 knockdown, retinal pigment epithelium cells, ARPE-19, cell proliferation, cell attachment, EMT, AKT signaling, β-catenin signaling, ERK signaling

## Abstract

Galectin-3 is a multifunctional protein that is associated with diseases of the chorioretinal interface, in which the retinal pigment epithelium (RPE) plays a central role in disease development and progression. Since galectin-3 can function extracellularly as well as intracellularly via different mechanisms, we developed an immortalized human RPE cell line (ARPE-19) with a knockdown for galectin-3 expression (ARPE-19/LGALS3^+/−^) using a sgRNA/Cas9 all-in-one expression vector. By Western blot analysis, a reduced galectin-3 expression of approximately 48 to 60% in heterozygous ARPE-19/LGALS3^+/−^ cells was observed when compared to native controls. Furthermore, ARPE-19/LGALS3^+/−^ cells displayed a flattened, elongated phenotype with decreased E-cadherin as well as enhanced N-cadherin and α-smooth muscle actin mRNA expression, indicating an epithelial–mesenchymal transition of the cells. Compared to wildtype controls, ARPE-19/LGALS3^+/−^ cells had significantly reduced metabolic activity to 86% and a substantially decreased proliferation to 73%. Furthermore, an enhanced cell adhesion and a diminished migration of immortalized galectin-3 knockdown RPE cells was observed compared to native ARPE-19 cells. Finally, by Western blot analysis, reduced pAKT, pERK1/2, and β-catenin signaling were detected in ARPE-19/LGALS3^+/−^ cells when compared to wildtype controls. In summary, in RPE cells, endogenous galectin-3 appears to be essential for maintaining the epithelial phenotype as well as cell biological functions such as metabolism, proliferation, or migration, effects that might be mediated via a decreased activity of the AKT, ERK1/2, and β-catenin signaling pathways.

## 1. Introduction

The retinal pigment epithelium (RPE) is fundamental for normal function and maintenance of visual phototransduction. For this, the monolayer RPE cells ensheathe the outer segments of photoreceptors by long microvilli on their apical surface to facilitate an intense interaction between both cells involving tissue homeostasis, the visual cycle, and phagocytosis [1]. On its basal side, RPE cells reside on the Bruch’s membrane, which separates them from the choriocapillaris. The integrity of this highly perfused capillary layer depends on the secretion of growth factors from the RPE. In turn, the choriocapillaris not only supplies the RPE with oxygen and essential nutrients but also the outer segments of the photoreceptors [1]. In view of its diverse functions, it is obvious that pathological changes in the RPE lead to a variety of diseases at the chorioretinal interface, such as retinal degenerations, age-related macular degeneration, or proliferative vitreoretinopathy [2].

In the past two decades, galectins have come into focus to modify pathologies of the chorioretinal interface. Galectins are a group of β-galactoside-binding proteins of mammals, which belong to the lectin family. Of the known 15 subtypes of galectins found in mammals, galectin-3 has a conserved carbohydrate-recognition domain at the C-terminus, like all galectins, to bind to carbohydrate moieties of glycoproteins, but it is unique since it contains an additional non-carbohydrate-binding moiety at the N-terminus, which enables galectin-3 to form pentamers [3]. Due to their cytosolic translation and unconventional secretion that bypasses the rough endoplasmic reticulum and the Golgi apparatus, galectins are located extracellularly as well as intracellularly [4]. While extracellular galectin-3 can promote clustering and functional modulation of specific glycoproteins as well as cell–cell interaction and cell adhesion via binding with its carbohydrate-recognition domain [5], it is intracellularly located in the cytosol and nucleus to modulate pre-mRNA processing, to inhibit apoptosis, or to modulate intracellular signaling pathways [5,6,7]. Due to its intra- or extracellular localization and its cell type as well as glycosylation-dependent binding, galectin-3, like all galectins, can have opposed functions.

Intriguingly, in the chorioretinal interface, conflicting data are available regarding the potential neuroprotective role of galectin-3 within the retina. For instance, in animal models for glaucoma or diabetic retinopathy, the reduced expression of galectin-3 mediated a protective effect on retinal neurons, most likely via decreased neuroinflammation [8,9,10]. Similarly, in a mouse model of light-induced photoreceptor damage, an inhibition of galectin-3 with the small molecule TD139 led to an increased survival of photoreceptor cells [11]. In contrast, in a mouse model with a general deficiency in pigment epithelium-derived factor, the additional inhibition of galectin-3 enhanced photoreceptor degeneration after light-induced retinal damage [12]. Furthermore, the light-induced damage of photoreceptors was increased in mice with a specific deletion of the galectin-3 gene in microglia cells, an effect that is most likely mediated via a reduced phagocytotic activity of microglia cells [13]. Further on, in a mouse model for wet age-related macular degeneration (AMD), a very common sight-threatening disease in elderly humans, the inhibition of galectin-3 led to smaller chorioretinal neovascularization and less subretinal fibrosis (CNV) [14]. In humans, increased levels for galectin-3 have been detected in proteins of the chorioretinal interface and in conditioned cell culture medium of RPE cells of patients suffering from AMD [15,16], pointing out that the function of galectin-3 involves the RPE.

In human RPE, we observed that galectin-3 in high amounts inhibits adhesion and spreading via a reduced cytoskeleton rearrangement [17]. In addition, incubation of human RPE cells with recombinant galectin-3 induces clustering of transmembrane proteins such as CD147 or integrin β1 and activates AKT and ERK signaling [18,19]. In human immortalized RPE (ARPE-19) cells, an enhanced expression of galectin-3 was observed following incubation in cell culture medium with high glucose, while the inhibition of the galectin-3 increase led to protective and anti-inflammatory effects [20]. In contrast, protective effects for galectin-3 were detected in ARPE-19 cells following UVA-induced damage [21]. Intriguingly, epithelial–mesenchymal transition (EMT) of RPE cells leads to an increased β1,6-N-glycosylation and to an enhanced binding of galectin-3 [22].

Since EMT of RPE cells occurs in diseases of the chorioretinal interface, such as AMD or proliferative vitreoretinopathy [23,24], and the functions of galectin-3 depend on several circumstances like its localization, the cell type, or glycosylation of the transmembrane proteins, we investigated the role of endogenous galectin-3 in ARPE-19 cells. To this end, we developed a galectin-3 knockdown ARPE-19 cell line using CRISPR/Cas9 technology and analyzed the effects of a reduced endogenous galectin-3 expression on the cell biological properties and common signaling pathways of RPE cells in vitro.

## 2. Results

### 2.1. Development ARPE-19 Cells with Reduced Galectin-3 Expression

**Figure 1:**
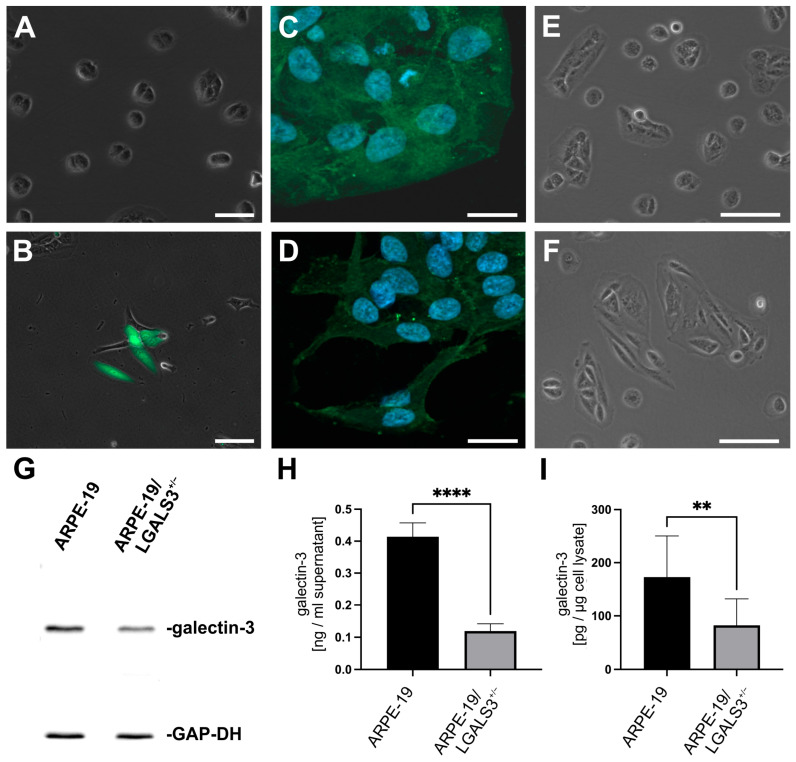
Development of galectin-3 knockdown ARPE-19 cells. (**A**,**B**) Merged phase-contrast and fluorescent imaging for GFP expression of ARPE-19 cells without (**A**) and with pCRISPR-LGALS3 transfection (**B**). (**C**,**D**) Immunostaining for galectin-3 of ARPE-19 (**C**) and ARPE-19/LGLAS3^+/−^ cells (**D**) after FACS and single-cell cultivation. (**E**,**F**) Phase-contrast image of ARPE-19 (**E**) and ARPE-19/LGLAS3^+/−^ cells (**F**). Magnification bar in (**A**,**B**) 100 µm; in (**C**,**D**) 20 µm, in (**E**,**F**) 100 µm; blue, Hoechst staining. (**G**) Western blot analysis for galectin-3 of ARPE-19 and ARPE-19/LGLAS3^+/−^ cells. (**H**,**I**) Galectin-3 concentration of conditioned cell culture medium (**H**) and cell lysate (**I**) from ARPE-19 and ARPE-19/LGLAS3^+/−^ cells after incubation in unsupplemented cell culture medium for 72 h. Mean ± SD; ** *p* < 0.01; **** *p* < 0.0001; n = 10.

To generate ARPE-19 cells lacking galectin-3 expression, the all-in-one sgRNA pCRISPR plasmid targeting human *LGALS3* (pCRISPR-LGALS3) was used. This plasmid expresses the Cas9 protein and a specific guide RNA, which assemble to form a highly specific endonuclease complex to target the galectin-3 gene. Additionally, the plasmid encodes a green fluorescent protein (GFP) for selection of transfected cells. Following transfection of ARPE-19 cells with pCRISPR-LGALS3, an intense GFP signal was observed in approximately 5 to 10% of the cells, indicating successful transduction of the vector (Figure 1B). To obtain ARPE-19 cells with a knockdown of galectin-3, fluorescence-activated cell sorting was performed, followed by single-cell cultivation. Several potential galectin-3-deficient ARPE-19 cell clones were tested by Western blot analysis. Of these, none had complete galectin-3 deficiency, while three clones showed reduced galectin-3 expression of approximately 60% by western blot analysis compared to wildtype controls (Figure 1G). To further confirm these observations, galectin-3 concentrations were measured in conditioned cell culture medium and cell lysates of ARPE-19/LGALS3^+/−^ cells and native controls following incubation in cell culture medium without supplements for 3 days. In line with our Western blot analyses, 173 ± 77 pg/µg total protein were detected in cell lysates of wildtype cells, while significantly lower levels of 82 ± 49 pg/µg total protein were observed (*p* < 0.01) in galectin-3 knockdown cells. In conditioned cell culture medium of ARPE-19/LGALS3^+/−^ cells, the galectin-3 concentration was 3.5-fold-lower (119 ± 23 pg/mL) when compared to that of normal controls (414 ± 43 pg/mL, *p* < 0.001), suggesting that the decreased expression of galectin-3 in ARPE-19/LGALS3^+/−^ cells affects its extracellular more than its intracellular concentrations. By immunohistochemistry, an intense staining for galectin-3 was detected in the cytoplasm and the nucleus of native ARPE-19 cells, which was substantially reduced in pCRISPR-LGALS3-treated cells (Figure 1C,D). In addition, we investigated the mRNA expression of galectin-3 in ARPE-19/LGALS3^+/−^ cells, which was 1.02 ± 0.15-fold when compared to native controls. Since classical CRISPR/Cas9 technology causes shifts in the reading frame of the mRNA, the observation of an unchanged galectin-3 mRNA expression is in line with a heterozygous inactivation of the gene. Since Western blot analysis, galectin-3 ELISA, and immunohistochemistry strongly suggest an inactivation of only one LGALS3 allele in ARPE-19 cells, these clones were designated hereinafter as heterozygous galectin-3 knockdown ARPE-19 cells (ARPE-19/LGALS3^+/−^).

Furthermore, besides reduced galectin-3 expression in ARPE-19/LGALS3^+/−^ cells, a flattened, round-to-oval or elongated, spindle-shaped phenotype was observed by phase-contrast microscopy in contrast to the typical cobblestone morphology of ARPE-19 cells, which were transfected, sorted, and single-cell cultured but had normal galectin-3 levels (ARPE-19/FACS) and native ARPE-19 cells (Figure 1E,F). To further confirm our morphological observations, the shortest and the longest diameters of ARPE-19 cells with a reduced galectin-3 expression as well as native controls were measured, and their quotient was formed. As expected, in ARPE-19/LGALS3^+/−^ cells, the quotient of the longest to the shortest axis was 2.1 ± 0.6, while in wildtype controls, it was 1.2 ± 0.2 (*p* < 0.01, n = 11).

### 2.2. Reduced Expression of Galectin-3 Decreases Viability of Immortalized RPE Cells In Vitro

To investigate the impact of galectin-3 on the viability of immortalized RPE cells, cell metabolism of native ARPE-19, ARPE-19/FACS, and ARPE-19/LGALS3^+/−^ cells with and without incubation with various concentrations of human recombinant (hr)-galectin-3 was analyzed by WST-1 assay.

**Figure 2:**
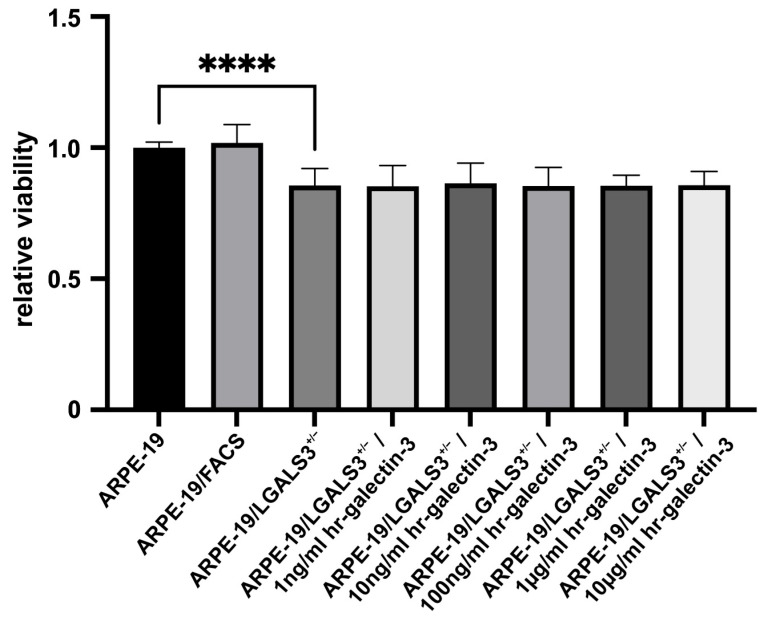
Reduced expression of galectin-3 decreases viability of immortalized RPE cells in vitro. WST-1 assay of ARPE-19, ARPE-19/FACS, and ARPE-19/LGALS3^+/−^ cells following incubation in cell culture medium without supplementation and various concentrations of hr-galectin-3 (hr-Gal3) for 72 h. Mean ± SD; **** *p* < 0.0001; n ≥ 28 of at least 4 independent experiments.

Following incubation for 72 h in cell culture medium without supplements, ARPE-19/LGALS3^+/−^ cells exhibited a significant reduction in WST-1 substrate turnover of approximately 15% (85.6 ± 6.4%) compared to native ARPE-19 cells (100.0 ± 21.2%; *p* < 0.001; Figure 2). Furthermore, the additional incubation of ARPE-19/LGALS3^+/−^ cells with various concentrations of hr-galectin-3 did not improve their viability, suggesting that intracellular galectin-3 mediates this effect on immortalized RPE cells.

### 2.3. Decreased Expression of Galectin-3 Declines Proliferation of Immortalized RPE Cells In Vitro

To analyze if expression of endogenous galectin-3 influences RPE cell proliferation, a BrdU ELISA on ARPE-19, ARPE-19/FACS, and ARPE-19/LGALS3^+/−^ cells was performed.

**Figure 3:**
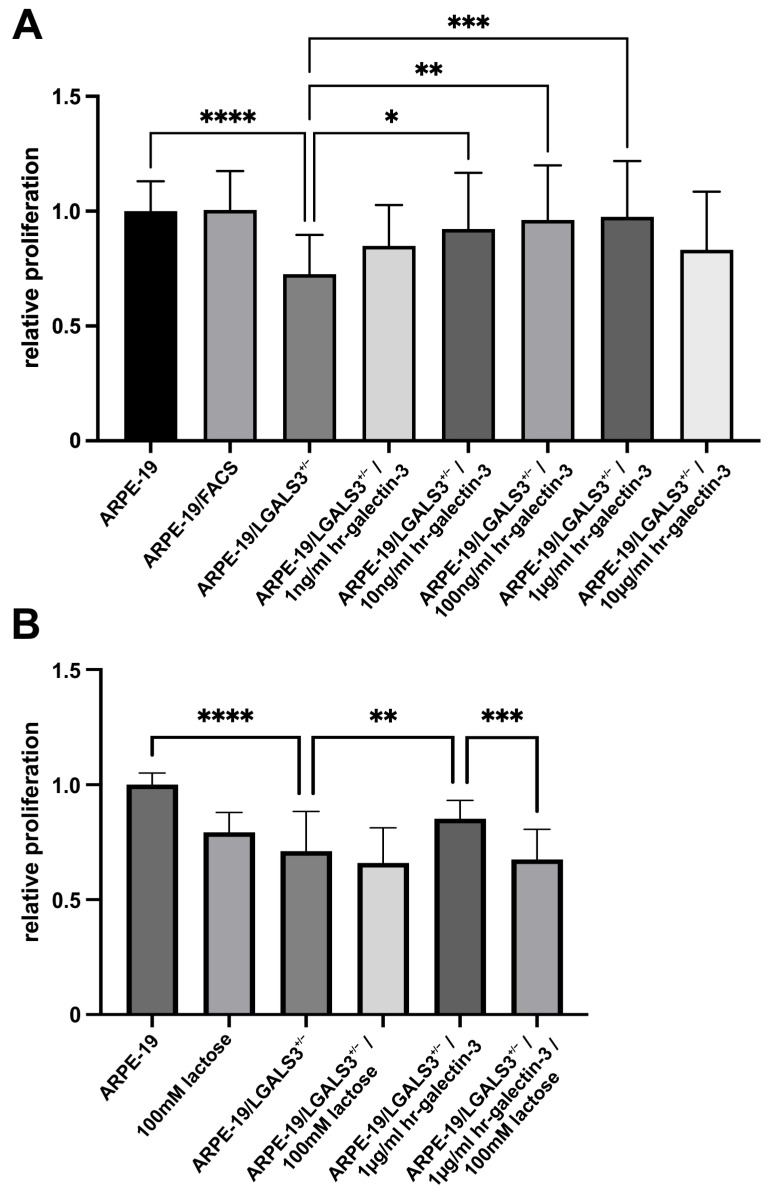
Decreased expression of galectin-3 declines proliferation of immortalized RPE cells in vitro. BrdU ELISA of ARPE-19, ARPE-19/FACS, and ARPE-19/LGALS3^+/−^ cells following incubation in cell culture medium without supplementation for 72 h with and without various concentrations of hr-galectin-3 (**A**) and/or an additional treatment with 100mM lactose (**B**). Mean ± SD; * *p* < 0.05; ** *p* < 0.01; *** *p* < 0.001; **** *p* < 0.0001; n ≥ 20 of at least 4 independent experiments.

After incubation for 72 h, no differences in the proliferation rate between ARPE-19 and ARPE-19/FACS cells were seen. In contrast, a significant decreased proliferation to 72.6 ± 17.1% was detected in ARPE-19/LGALS3^+/−^ when compared to native ARPE-19 (Figure 3A; Figure S1 (boxplot); 100.0 ± 13%; *p* < 0.0001). Intriguingly, the decreased proliferation of galectin-3 knockdown ARPE-19 cells could be rescued by the addition of hr-galectin-3 in a dose-dependent manner (Figure 3A). To further analyze if hr-galectin-3 mediates its effects via extracellular β-galactoside binding, the cells were additionally incubated with 100 mM lactose. Intriguingly, treatment of ARPE-19/LGALS3^+/−^ and native ARPE-19 cells with 100 mM lactose led to a reduced proliferation when compared to controls. As described above, 1 µg/mL hr-galectin-3 could significantly enhance proliferation of ARPE-19/LGALS3^+/−^ (85.2 ± 8.0%; *p* < 0.01) compared to untreated controls (Figure 3B; 71.1 ± 17.3%). After an additional incubation of galectin-3 knockdown cells with 100 mM lactose, the hr-galectin-3-mediated effect could be reversed to 67.5 ± 13.1% (Figure 3B; *p* < 0.001), strongly indicating that endogenous galectin-3 promotes proliferation of immortalized RPE cells at least in part via extracellular mechanisms.

### 2.4. Galectin-3 Promotes Migration of Immortalized RPE Cells In Vitro

To investigate the effect of endogenous galectin-3 on the migration of immortalized RPE cells, a scratch migration assay was performed.

**Figure 4:**
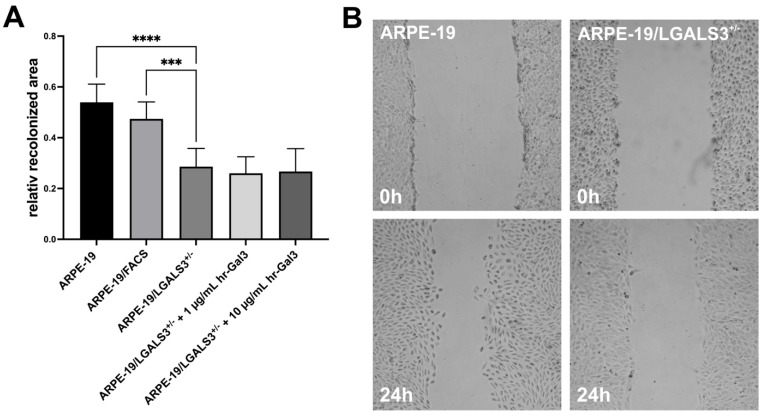
Decreased galectin-3 expression reduces migration of ARPE-19 cells in vitro. Quantification (**A**) and representative images (**B**) of scratch migration assay of native ARPE-19, ARPE-19/FACS, and ARPE-19/LGALS3^+/−^ cells following incubation with and without hr-galectin-3 in cell culture medium without supplements for 24 h. (**A**) For quantification, the recolonized area after 24 h was calculated and plotted as the relative recolonized area. (**B**) Representative images of native ARPE-19 (left panel) and ARPE-19/LGALS3^+/−^ cells (right panel) immediately after scratching (0 h, upper row) and after incubation for 24 h (lower panel). Mean ± SD; *** *p* < 0.001; **** *p* < 0.0001; n ≥ 10 of 3 independent experiments.

After incubation for 24 h, an area of 62.6 ± 7.9% of the scratched area was covered by native ARPE-19 cells, while the recolonized area was only 28.6 ± 7.2% in ARPE-19/LGALS3^+/−^ cells (Figure 4; *p* < 0.001), demonstrating that a lack of endogenous galectin-3 decreases velocity of galectin-3 knockdown ARPE-19 cells. Furthermore, the additional treatment of ARPE-19/LGALS3^+/−^ cells with 1 and 10 µg/mL hr-galectin-3 had no effect on cell migration of galectin-3 knockdown cells (Figure 4), strongly suggesting that endogenous, intracellular galectin-3 is essential for cell migration of immortalized RPE cells.

### 2.5. Expression Enhances Cell Attachment in Immortalized RPE Cells In Vitro

To investigate if endogenous galectin-3 can modulate cell adhesion, a cell adhesion assay was performed.

**Figure 5:**
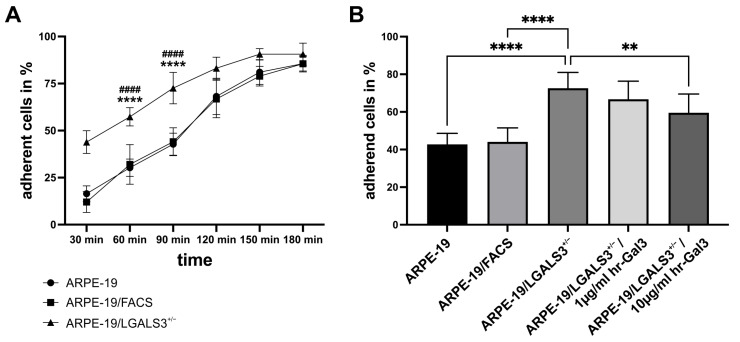
Reduced expression of endogenous galectin-3 enhances cell attachment of immortalized RPE cells in vitro. (**A**) Cell adhesion of ARPE-19, ARPE-19/FACS, and ARPE-19/LGALS3^+/−^ cells was quantified 30, 60, 90, 120, 150, and 180 min after seeding and plotted as the relative number of adherent cells. Mean ± SD; n = 12 for each group of 3 independent experiments; * comparison of ARPE-19 versus ARPE-19/LGALS3^+/−^ cells; # comparison of ARPE-19/FACS versus ARPE-19/LGALS3^+/−^ cells; **** *p* < 0.001; #### *p* < 0.001. (**B**) Relative number of adherent ARPE-19, ARPE-19/FACS, and ARPE-19/LGALS3^+/−^ cells 90 min after seeding and incubation with various concentrations of hr-galectin-3, which was added immediately before seeding. Mean ± SD; n = 15 for each group of 3 independent experiments; ** *p* < 0.01; **** *p* < 0.0001.

After incubation for 30 min after seeding, significantly more ARPE-19/LGALS3^+/−^ cells (43.9 ± 9.2%) were attached to the bottom of the culture dish when compared to native ARPE-19 cells (12 ± 5.6%; *p* < 0.001) and ARPE-19/FACS cells (12 ± 5.6%; *p* < 0.001, Figure 5A). During prolonged incubation, the number of adhered cells increased in all groups. However, the number of adherent ARPE-19/LGALS3^+/−^ cells was significantly higher at up to 150 min after seeding, strongly indicating that galectin-3 reduces adhesion of immortalized RPE cells.

Furthermore, to analyze if the adhesive effects of decreased galectin-3 expression could be reversed, attachment of ARPE-19/LGALS3^+/−^ cells was investigated in the presence of hr-galectin-3, which was added immediately before seeding. Similarly, only 42.8 ± 5.9% of wildtype ARPE-19 cells were adhered 90 min after seeding, while 72.6 ± 8.4% of the galectin-3 knockdown cells were attached (*p* < 0.0001). In contrast, the additional incubation with 10 µg/mL hr-galectin-3 led to a 13.1% reduction in the number of adhered ARPE-19/LGALS3^+/−^ cells (59.5 ± 10%; *p* < 0.01; Figure 5B) when compared to untreated galectin-3 knockdown cells. Since the enhanced adhesion of galectin-3 knockdown cells could be rescued by the addition of hr-galectin-3, it is most likely that endogenous galectin-3 diminishes adhesion of immortalized RPE cells via an extracellular function.

### 2.6. Reduced Expression of Galectin-3 Promotes Epithelial-to-Mesenchymal Transition of Immortalized RPE Cells In Vitro

Since we observed an elongated, spindle-shaped phenotype of ARPE-19/LGALS3^+/−^ cells, we investigated whether the absence of galectin-3 promotes epithelial-to-mesenchymal transition (EMT) by immunofluorescent staining and real-time RT-PCR.

**Figure 6:**
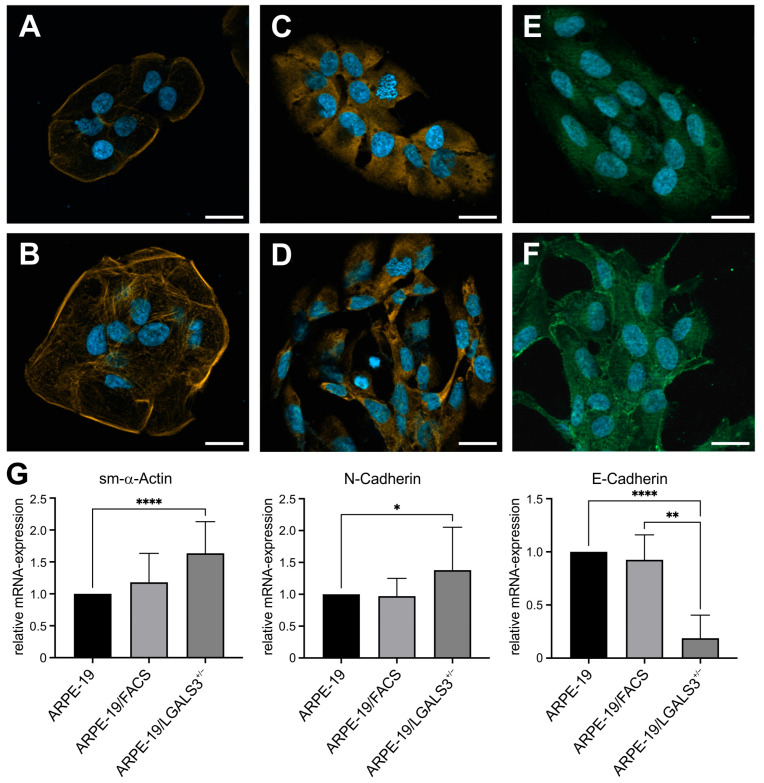
Lack of endogenous galectin-3 promotes epithelial-to-mesenchymal transition of immortalized RPE. (**A**–**F**) Phaloidin staining ((**A**,**B**) orange) as well as immunofluorescent staining for sm-α-actin ((**C**,**D**) orange) and N-cadherin (**E**,**F**) green) of ARPE-19 (**A**,**C**,**E**) and ARPE-19/LGALS3^+/−^ cells (**B**,**D**,**F**). Scale bar, 20 µm; blue, DAPI staining. (**G**) Real-time rt-PCR for sm-α-actin, E-cadherin, and N-cadherin mRNA expression of ARPE-19, ARPE-19/FACS, and ARPE-19/LGALS3^+/−^ cells. Mean ± SD; n ≥ 7 of 5 independent experiments; * *p* < 0.05; ** *p* < 0.01; **** *p* < 0.0001.

In native ARPE-19 cells, phalloidin labeling, which stains F-actin, was predominately located at cell membrane sections that had no contact with neighboring cells. In addition, only a few stress fibers spanning across the cells were observed (Figure 6A). In contrast, in ARPE-19/LGALS3^+/−^ cells, the signal for phalloidin was much more pronounced, and the number of stress fibers was higher when compared to wildtype controls (Figure 6B) suggesting a more mesenchymal phenotype of galectin-3 knockdown cells. In line, the fluorescent signal for smooth muscle (sm)-α-actin and N-cadherin, both markers for EMT, was increased in ARPE-19/LGALS3^+/−^ cells (Figure 6C–F) when compared to native controls, again indicating an EMT of these cells.

To further confirm our histological observations, real-time rt-PCR for E-cadherin, N-cadherin, and sm-α-actin mRNA was performed. In ARPE-19/LGALS3^+/−^ cells, the mRNA expression of both N-cadherin and sm-α-actin was moderately but significantly increased to 137.9 ± 14.6% (* *p* < 0.05) and 163.5 ± 10.8% (*p* < 0.0001), respectively, when compared to native ARPE-19 cells (Figure 6G). In contrast, the mRNA expression for E-cadherin, a marker for an epithelial phenotype, was substantially decreased to 18.7 ± 8.1% in galectin-3 knockdown cells when compared to native ARPE-19 cells (* *p* < 0.01, Figure 6G). Overall, our histological as well as mRNA data strongly suggest that endogenous galectin-3 is required to maintain the epithelial phenotype of immortalized RPE cells in vitro.

### 2.7. Endogenous Galectin-3 Is Essential for Maintaining of AKT, ERK, and Wnt/β-Catenin Signaling in Immortalized RPE Cells In Vitro

Since we found that a reduced expression of galectin-3 leads to significant changes in the cell biology of immortalized RPE cells, such as reduced proliferation and migration, we analyzed whether these effects could be mediated via a modulation of AKT, ERK, or β-catenin signaling pathways.

**Figure 7:**
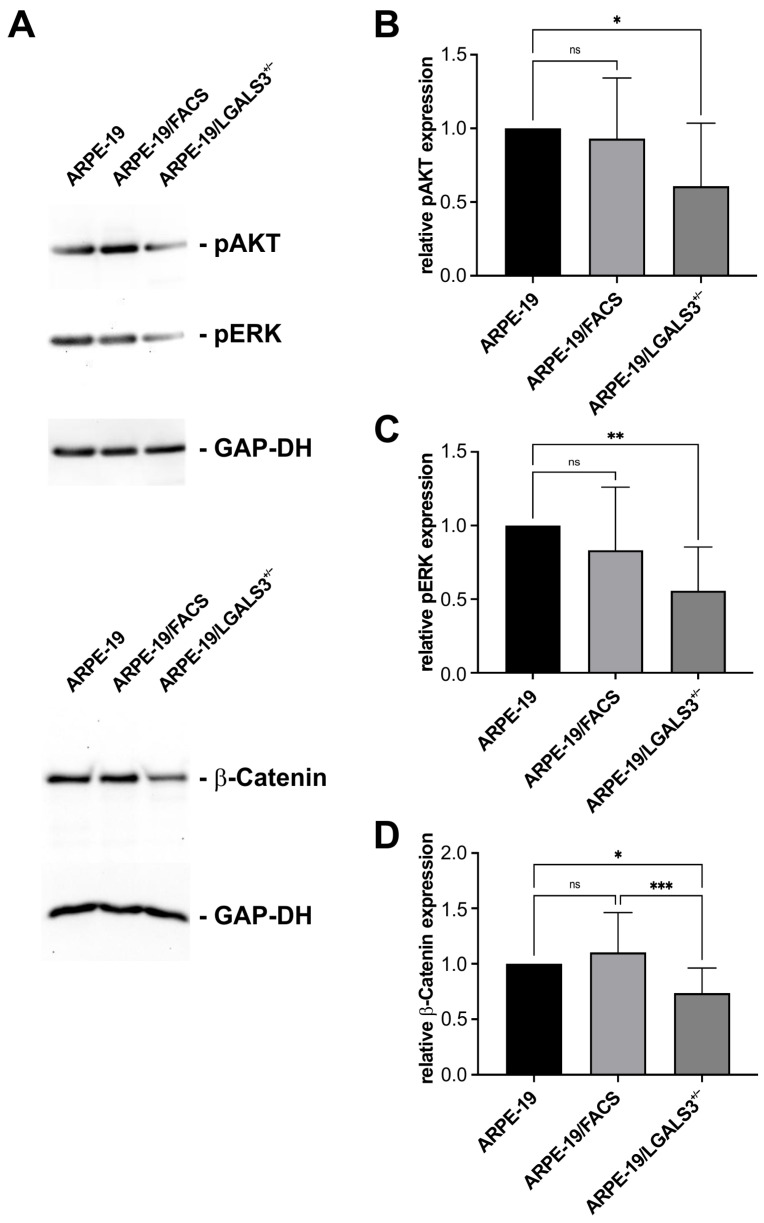
Endogenous galectin-3 expression maintains basal pAKT, pERK, and β-catenin signaling in immortalized RPE cells. Western blot analysis (**A**) and densitometry for pAKT (**B**), pERK (**C**), and β-catenin (**D**) expression of ARPE-19, ARPE-19/FACS, and ARPE-19/LGALS3^+/−^ cells. Mean ± SD; n ≥ 6 of 6 independent experiments; * *p* < 0.05; ** *p* < 0.01; *** *p* < 0.001; ns, not significant.

By Western blot analyses, specific bands for pAKT, pERK, and β-catenin were detected in native controls, which had a similar intensity like that of FACS/ARPE-19 cells. In contrast, in ARPE-19/LGALS3^+/−^ cells, the signals for pAKT, pERK, and β-catenin were significantly lower than in both control groups (Figure 7A). By densitometry, the relative pAKT, pERK, and β-catenin expression in ARPE-19/LGALS3^+/−^ cells was 60.1 ± 11.9% (*p* < 0.05), 55.5% ± 13.6% (*p* < 0.01), and 73.4 ± 0.6% (*p* < 0.05), respectively, when compared to the ARPE-19 controls, while no relevant differences were seen between FACS/ARPE-19 and native controls (Figure 7B–D). Overall, our data strongly suggest that endogenous galectin-3 is required to maintain endogenous AKT, ERK, and β-catenin signaling in immortalized RPE cells in vitro.

## 3. Discussion

We conclude that endogenous galectin-3 is essential for various cell biological functions and the maintenance of the epithelial phenotype of human RPE cells in vitro. Our conclusions rest upon (1) the finding that the decreased expression of galectin-3 leads to a reduced viability and proliferation of immortalized RPE cells, (2) the observation of a decreased migration of galectin-3 knockdown cells, (3) the result of an enhanced attachment of ARPE-19/LGALS3^+/−^ cells, (4) the discovery of an intensified epithelial-to-mesenchymal transition in galectin-3 knockdown RPE cells, (5) and finally the finding of a decreased activity of pAKT, pERK1/2, and β-catenin signaling pathways in ARPE-19/LGALS3^+/-^ cells.

To analyze the endogenous role of galectin-3 in human RPE cells, we generated an immortalized human RPE cell line with a reduced expression of galectin-3 by CRISPR/Cas9 technology. Intriguingly, none of the investigated potential cell clones had a complete lack of galectin-3 expression, but rather only cell lines with a decreased expression of approximately 60% by Western blot analysis and 48% by ELISA were identified in cellular lysates. Depending on the measurement accuracy of the test procedure and knowing the increased variations in gene expression when only one allele is active [25], these observations suggest an average reduction in galectin-3 expression by about half and hence a haploid deficiency for galectin-3 in these cells. In contrast, in conditioned cell culture medium from ARPE-19/LGALS3^+/-^ cells, the galectin-3 level was approximately 3.5-fold-less compared to normal cells, indicating an additional substantially decreased secretion of the protein in galectin-3 knockdown ARPE-19 cells. Incomplete genome editing, which can lead to monoallelic targeting and gene modification, is common in CRISPR/Cas9 applications and in turn allows the unedited allele to retain its function [26,27]. However, in a recent work targeting the galectin-1 gene in immortalized RPE cells with the same vector system, a homozygous disruption of the gene was achieved [28]. Therefore, it is tempting to speculate if a homozygous deletion of the galectin-3 gene in combination with the generation procedure, including single-cell cultivation, could be lethal for human immortalized RPE cells.

By various cell biological assays, we observed a decreased viability, proliferation, and migration and an enhanced attachment of immortalized RPE cells with a decreased expression of galectin-3. Intriguingly, by adding human recombinant galectin-3, only the reduced proliferation and the enhanced attachment of ARPE-19/LGALS3^+/−^ cells could be reversed, while exogenous galectin-3 had no effects on their reduced viability and migration. First, the rescue of the reduced proliferation and the increased attachment of ARPE-19/LGALS3^+/−^ cells by exogenous galectin-3 clearly points out that the phenotype of these cells is galectin-3-dependent. Since the proliferative effect of hr-galectin-3 on galectin-3 knockdown cells could be reversed by lactose and the endogenous extracellular level for galectin-3 in these cells are substantially decreased, it is most likely that these effects are mediated via an extracellular β-galactoside-binding of galectin-3. However, the missing reversing effect of exogenous galectin-3 for cell viability and migration of ARPE-19/LGALS3^+/−^ cells could depend on the intracellular functions of galectin-3 for these cell biological properties, which were not affected by the exogenous addition of recombinant galectin-3. In line with our observations, in astrocytoma or nasopharyngeal carcinoma cells, the knockdown of galectin-3 by si- or sh-RNA led to a reduced viability and migration [29,30]. Since PI3K/AKT signaling plays an essential role in aerobic glycolysis via phosphorylating different nutrient transporters and metabolic enzymes [31], and ERK signaling plays a central role for cell migration [32], which both have a reduced activity in our knockdown cells, it is most likely that the reduced viability and mobility of ARPE-19/LGALS3^+/−^ cells could be caused by decreased AKT and ERK signaling in these cells.

By real-time RT-PCR and immunocytochemical staining, an increased expression of smooth muscle α-actin and N-cadherin, as well as a decreased level of E-cadherin, were observed in immortalized human galectin-3 knockdown ARPE-19 cells, suggesting an epithelial–mesenchymal transition of these cells. EMT is a process that is involved in various developmental, regenerative, and pathological conditions and is accompanied with a loss of cell polarity and cell–cell adhesion properties to acquire a mesenchymal phenotype, which is associated with an increased motility and invasiveness [33,34]. Increased level of galectin-3 has been reported to promote EMT in several cell types following active stimulation or in tumor biology via enhanced pAKT, pERK1/2, or β-catenin signaling [35]. In contrast, in a subgroup of breast cancers, a loss of galectin-3 expression was associated with EMT [36], indicating a differentiated role for galectin-3 in this process. Similarly in our ARPE-19/LGALS3^+/−^ cells, we additionally found decreased pAKT, pERK1/2, and β-catenin signaling as well as reduced mobility of cells, arguing against a canonical pathway leading to EMT in galectin-3 knockdown cells. Furthermore, in RPE cells, EMT not only depends on the activation of specific EMT pathways and the expression of galectin-3 but also on the glycosylation of galectin-3-binding partners [22]. In addition, galectin-3 interacts with various transcription factors such as the thyroid transcription factor 1 (TTF-1) [37]. Since TTF-1 can inhibit TGF-β-mediated EMT and restore the epithelial phenotype in lung adenocarcinoma cells [38], it is tempting to speculate that the decreased expression of galectin-3 leads to a reduced activity of transcription factors, such as TTF-1, which are essential to maintain the epithelial phenotype of RPE cells.

By Western blot analyses, we observed a decreased expression of pAKT, pERK1/2, and β-catenin in galectin-3 knockdown RPE cells. For the interaction of pAKT and pERK1/2 with galectin-3, several reports indicate that galectin-3 enhances their signaling, while its knockdown leads to a decreased phosphorylation of both proteins [30,39,40], which is in line with our observations. Furthermore, similar results were detected for the activity of β-catenin signaling, which depends on the activity of glycogen synthase kinase 3β (GSK-3β), a central component of the β-catenin destruction complex [41]. Within this signaling network, pAKT phosphorylates and in turn inactivates the GSK-3β, which in turn leads to an accumulation and translocation of β-catenin into the nucleus [41]. Overall, the decreased expression of galectin-3 in RPE cells led to reduced AKT, ERK1/2, and β-catenin signaling, which in turn could be the reason for the diminished proliferation and migration of these cells.

In summary, endogenous galectin-3 in RPE cells promotes cell viability, proliferation, as well as motility and reduces cell adhesion. Since these cell biological properties are hallmarks of diseases of the chorioretinal interface, it is tempting to speculate that the inhibition of galectin-3 in RPE cells could be a potential option for the treatment of these diseases, including proliferative vitreoretinopathy, diabetic retinopathy, or wet age-related macular degeneration.

## 4. Methods

### 4.1. Cell Culture

ARPE-19 cells were purchased from ATTC (Manassas, VA, USA) and cultured under standard conditions in an incubator at 37 °C and 5% CO_2_. Cell culture medium (DMEM/HAM’s F12 medium, Bio&Sell, Nuernberg, Germany), which contained 10% fetal calf serum, 50 μg/mL penicillin, and 50 μg/mL streptomycin (Merck, Darmstadt, Germany), was changed every other day. Semiconfluent ARPE-19 cells were detached with versene solution with 0.05% trypsin (both from Thermo Fisher, Waltham, MA, USA) for subcultivation. For immunohistochemistry, ARPE-19 cells were seeded and grown on glass coverslips. Before the experiment the cells were incubated in cell culture medium without FCS for 24h. For rescue experiments, cells were incubated with 1ng/mL to 10 μg/mL human recombinant (hr)-galectin-3.

### 4.2. Isolation of Human Recombinant Galectin-3

Human recombinant (hr) galectin-3 was isolated from transfected E. coli as previously described [18]. In brief, human recombinant galectin-3 expressing E. coli were cultured at 20 °C in ZYM 5052 autoinduction medium and 100 μg/mL kanamycin (Merck) [42]. Following centrifugation, the cells were resuspended in 30 mL lysis buffer (20 mM Tris-HCl, 150 mM NaCl, 10 mM MgSO4, 10 μg/mL DNase1, 1 mM AEBSF.HCl, 0.03% (*v*/*v*) CHAPS, 1 mg/mL lysozyme, pH 7.5) and lysed by sonication. After an additional centrifugation with 40,000× *g* and filtration (0.2 μm) of the lysates, supernatants were loaded on lactose-agarose columns, which were equilibrated in buffer A (20 mM Tris-HCl, 150 mM NaCl, 0.03% (*v*/*v*) CHAPS, pH 7.5). After 3 washes with 25 mL buffer A, bound proteins were eluted two times with 5 mL buffer A containing 0.2 M β-lactose. Following dialyzation overnight at 4 °C against 1× PBS, the isolated proteins were filtered (0.2 μm) and stored at 4 °C. The concentration of the isolated galectin-3 protein was determined by measuring the absorbance at 280 nm.

### 4.3. Transfection

Galectin-3 knockdown ARPE-19 cells were generated using the all-in-one sgRNA pCRISPR plasmid against human *LGALS3* (pCRISPR-LGALS3; HCP301784-CG04-3-Bc, Genecopoeia, Rockville, MA, USA). Besides its expression of a specific gRNA and the CAS9 protein for editing of the galectin-3 gene, this vector additionally transcribes a neomycin resistance gene as well as a green fluorescent protein (GFP) for selection of transfected cells. Prior to transfection, ARPE-19 cells were seeded in a 24-well plate (Sarstedt, Nuembrecht, Germany) and cultured until confluence of approximately 60%. Transfection of the cells with up to 1 µg of pCRISPR-LGALS3 plasmid DNA was performed with Lipofectamine 3000 (Thermo Fisher) in accordance with the manufacturer’s recommendations. After incubation of the cells at 37 °C for 2 h, the transfection medium was removed and supplemented with DMEM/Ham’s-F12 cell culture medium containing 10% fetal calf serum was added for an additional 24 h. To confirm the successful transformation the expression of GFP was investigated using the inverted fluorescence microscope Axio Observer 7 (Zeiss, Oberkochen, Germany).

### 4.4. Fluorescence-Activated Cell Sorting

To select potential galectin-3 knockdown cells, transformed ARPE-19 cells were sorted and single-cell cultured using the FACSAria^TM^ III Cell Sorter (BD Life Sciences, San Jose, CA, USA) according to the manufacturer’s recommendations. In brief, cells with a 1000-fold-higher GFP signal than native ARPE-19 cells were considered as positively transformed and were seeded into a single well of a 96-well plate (Sarstedt) containing DMEM/HAM’s F12 cell culture medium, which was supplemented with 10 ng/mL epithelial growth factor, 20 ng/mL fibroblastic growth factor-2 (both from Biolegend, San Diego, CA, USA), 20% FCS, 50 μg/mL penicillin, and 50 μg/mL streptomycin. After 48 h, the cell culture medium was replaced, and cell cultivation was continued under standard conditions as described above.

### 4.5. Cell Proliferation and Cell Viability

Cell proliferation was analyzed using the 5-bromo-2-deoxyuridine (BrdU) ELISA (Merck) in accordance with the manufacturer’s recommendations.

In brief, approximately 4000 cells per well were seeded into a 96-well plate and incubated for 24 h under standard conditions to ensure complete cell adherence. Following an additional incubation with BrdU-labeling solution and various concentrations of hr-galectin-3 in unsupplemented cell culture medium for 72 h, cells were fixed and incubated with anti-BrdU antibodies. The amount of BrdU incorporation into the DNA was quantified by absorbance measurement at a wavelength of 450 nm and a reference at 690 nm on the SpectraMax 190 ELISA reader (Molecular Devices, San Jose, CA, USA).

Cell viability was analyzed using the water-soluble tetrazolium dye WST-1 (Merck). To this end, 10,000 cells per well were seeded into a 96-well plate and cultured in supplemented cell culture medium for 24 h to ensure full cellular recovery. Before incubation for 72 h, various concentrations of hr-galectin-3 in cell culture medium without supplements were added. Following incubation in WST-1 containing cell culture medium for up to 30 min, the absorbance of each well was measured at a wavelength of 450 nm and a reference at 690 nm on a SpectraMax 190 ELISA reader (Molecular Devices).

### 4.6. Scratch Migration Assay

Cells were seeded in a 6-well-plate and cultured until confluency. A 100 μL pipette tip was used to create a wound area in the cellular monolayer, followed by two washes with 1× PBS to remove cell debris. Immediately after wounding as well as 24 h thereafter, the cell-free area was documented using an inverted Axio Observer 7 microscope (Zeiss, Oberkochen, Germany). The cell-free area before and after incubation for 24 h was quantified using the ZEN software Blue edition 3.0 (Zeiss) and plotted as the relative recolonized area.

### 4.7. Cell Adhesion Assay

For the analysis of cell attachment, approximately 1000 cells/cm2 were seed in a 6-well plate. Images were taken after an incubation for 30, 60, 90, 120, and 150 min using an inverted Axio Observer 7 microscope. For rescue experiments, 1 and 10 µg/mL of hr-galectin-3 were added immediately before seeding. The number of adherent and total cells was quantified and expressed as the relative number of attached cells.

### 4.8. Galectin-3 ELISA

For measurement of the galectin-3 amount in conditioned cell culture medium and cell lysates, a galectin-3 ELISA was used in accordance with the manufacturer’s instructions (Human Galectin-3 ELISA Kit, Proteintech, Rosemont, IL, USA).

For this, semiconfluent ARPE-19/LGALS3^+/−^ and native controls were incubated with cell culture medium without supplements for 3 days. Approximately 50 µL of conditioned cell culture medium and 2 µg of the cell lysate were diluted with Sample Dilutant to a final volume of 100 µL, transferred into the wells of the microtiter plate, and incubated for 2 h. Following four washes with wash buffer, 100 µL per well of diluted HRP-conjugated detection antibodies were added and incubated for another 40 min. After an additional four washings, 100 µL of TMB substrate were added to each well and incubated for 10 min. Before measuring the absorption at a wavelength of 450 nm and a reference wavelength of 690 nm with a SpectraMax 190 ELISA reader (Molecular Devices), 100 µL of the stop solution were added to each well. The concentration of the specimens was calculated using the SoftMax Pro software (Version 6.5.1, Molecular Devices).

### 4.9. Immunohistochemistry

Cells were fixed with 4% paraformaldehyde for 10 min. After 3 washes for 5 min with 0.1 M phosphate buffer (0.1 M Na_2_HPO_4_ × 2 H_2_O, 0.1 M NaH_2_PO_4_ × H_2_O, pH 7.4), the cells were blocked with 3% bovine serum albumin with 0.1% Triton X-100 in 0.1 M phosphate buffer for 30 min and incubated overnight with 1:100 rat α-galectin-3 (Biolegend), 1:100 mouse smooth muscle-α-actin (both Santa Cruz, Dallas, TX, USA), 1:100 mouse α-N-cadherin (Thermo Fisher), and 1:100 phalloidin Alexa Fluor 555 (Thermo Fisher) in 0.3% bovine serum albumin and 0.01% Triton X-100 in 0.1 M phosphate buffer at 4 °C. Specimens were then washed again 3 times for 10 min each with 0.1 M phosphate buffer and incubated with 1:1000 diluted monkey anti-rat antibody Alexa Fluor 488 or goat anti-mouse Alexa Fluor 488 (both from Thermo Fisher) in 1:10 diluted blocking solution for 1 h at room temperature. Following nuclear staining with Hoechst 33,342 (Thermo Fisher), all specimens were washed again 3 times with 0.1 M phosphate buffer and mounted with the ProLong glass antifade mounting medium (Thermo Fisher). Immunofluorescence staining was analyzed by an Axio Observer 7 fluorescence microscopy with an Apotome 2 module (Zeiss, Oberkochen, Germany) and documented using the ZEN software Blue edition 3.0 (Zeiss).

### 4.10. Protein Preparation and Western Blot Analysis

ARPE-19/LGALS3^+/−^, ARPE-19/FACS, and ARPE-19 cells were lysed in RIPA buffer containing protease and phosphatase inhibitors (Complete; Merck). Up to 15 µg of the total protein per sample was loaded onto a 10% SDS page and transferred onto a PVDF membrane (Roche, Mannheim, Germany) by semidry blotting following electrophoresis. After blocking with 3% skim milk powder in 1× PBST, membranes were incubated with 1:1000 diluted rat α-galectin-3 (Biolegend), 1:1000 rabbit α-pAKT (R&D System, Minneapolis, MN, USA), 1:1000 rabbit α-pERK1/2 (R&D System), and 1.000 rabbit α-β-catenin antibodies (Abcam, Cambridge, UK) in 1× PBS with 0.3% skim milk powder and 0.1% Tween 20 at 4 °C overnight. After 3 washes with 1× PBS for 10 min each, the membranes were incubated with 1:1000 alkaline phosphatase (AP) conjugated α-rat or α-rabbit antibodies (Jackson ImmunoResearch Europe, Ely, UK) in 1× PBS with 0.3% skim milk powder and 0.1% Tween 20 for 1 h. As a loading control, blots were hybridized with 1:5000 mouse α-GAP-DH (Merck) antibodies in 1× PBS with 0.3% skim milk powder and 0.1% Tween 20 for 1 h at room temperature, followed by an additional incubation with 1:1000 AP-conjugated α-mouse antibodies (Dianova, Hamburg, Germany) in 1× PBS with 0.3% skim milk powder and 0.1% Tween 20 at room temperature for 1 h. For visualization, membranes were visualized with CDP-Star substrate according to the manufacturer’s instructions (CDP-Star, Thermo Fisher) and documented with an iBrightCL1000 Imaging System (Thermo Fischer).

### 4.11. RNA Isolation, cDNA Synthesis and Real-Time RT-PCR

For mRNA expression analyses, ARPE-19/LGALS3^+/−^, ARPE-19/FACS, and ARPE-19 cells were lysed in TriFast (VWR, Leuven, Belgium), and total RNA was isolated in accordance with the manufacturer’s instructions. RNA Concentration and the OD260/OD280 ratio were measured using the Biophotometer (Eppendorf, Hamburg, Germany). Only total RNA with a 260/280 ratio between 1.8 and 2.0 was used for first-strand cDNA synthesis, which was performed according to the manufacturer’s recommendations (iScript gDNA CLR cDNA Synthesis Kit, Bio-Rad, Hercules, CA, USA). Quantitative real-time RT-PCR analyses were performed on a Bio-Rad CFX Real-Time PCR Detection System using the 2× iTaq Univer SYBR Green SMX mix (Bio-Rad) in accordance with the manufacturer’s protocol. PCR was performed in a final volume of 15 µL, which contained 7.5 µL 2× iTaq Univer SYBR Green SMX mix, 1 µL cDNA, and 0.16 µL of primer mix (1 µM each, Thermo Fisher). The temperature profile was 10 s denaturation at 95 °C and 1 min annealing and extension at 60 °C for 40 cycles, after initial activation at 95 °C for 3 min. As negative control, RNA that had not been reversed-transcribed was used. All PCR primers were designed to span exon–intron boundaries and were purchased from Thermo Fisher (Table 1). For relative quantification, the reference gene GNB2L was used. Results were analyzed using the ΔΔCT method which is included in the Bio-Rad CFX manager software (version 3.1).

### 4.12. Statistical Analysis

All calculations and statistical analyses were performed using Prism version 10 (GraphPad Software, Bosten, MA, USA) and EXCEL 365 version 16 (Microsoft, Redmond, WA, USA). For all data, the mean and the standard deviation (SD) were calculated and shown. For comparison of two groups, a Student’s t-test was performed, and for more than two groups, a one-way ANOVA was performed. For data that meet the assumption of homogeneity of variances, a least significant difference (LSD) post hoc test was performed, and for data not meeting the criteria, a Games Howell post hoc test was performed. All *p* values less than 0.05 were considered statistically significant.

## Figures and Tables

**Table 1:** Primers used for real-time rt-PCR.

Gene	Accession No.	Sequence	Product Size
E-cadherin	NM_004360	5’-cccgggacaacgtttattac-3’ 5’-gctggctcaagtcaaagtcc-3’	71 bp
*LGALS3*	NM_002306.3	5’-cttctggacagccaagtgc-3’ 5’-aaaggcaggttataaggcacaa-3’	94 bp
N-cadherin	NM_001308176	5’-ggtggaggagaagaagaccag-3’ 5’-ggcatcaggctccacagt-3’	72 bp
sm-α-actin	NM_001613	5’-ctgaagtacccgatagaacatgg-3’ 5’-ttgtagaaagagtggtgccagat-3’	77 bp
GNB2L	NM_006098	5’-ctacaatgatctttccctctaaatcc-3’ 5’-cctaaccgctactggctgtg-3’	72 bp

## Data Availability

The data presented in this study are available on request from the corresponding author.

## Supplementary Materials

The following supporting information can be downloaded at: https://www.mdpi.com/article/10.3390/ijms26157622/s1.
