# Supplementary material for: Role of Endogenous Galectin-3 on Cell Biology of Immortalized Retinal Pigment Epithelial Cells In Vitro
Source: Int J Mol Sci. 2025 Aug 6;26(15):7622. doi: 10.3390/ijms26157622 (PMC12347958; doi:10.3390/ijms26157622)
Supplement: Supplementary file 1 [file ijms-26-07622-s001.zip › ijms-3730903-supplementary.pdf]

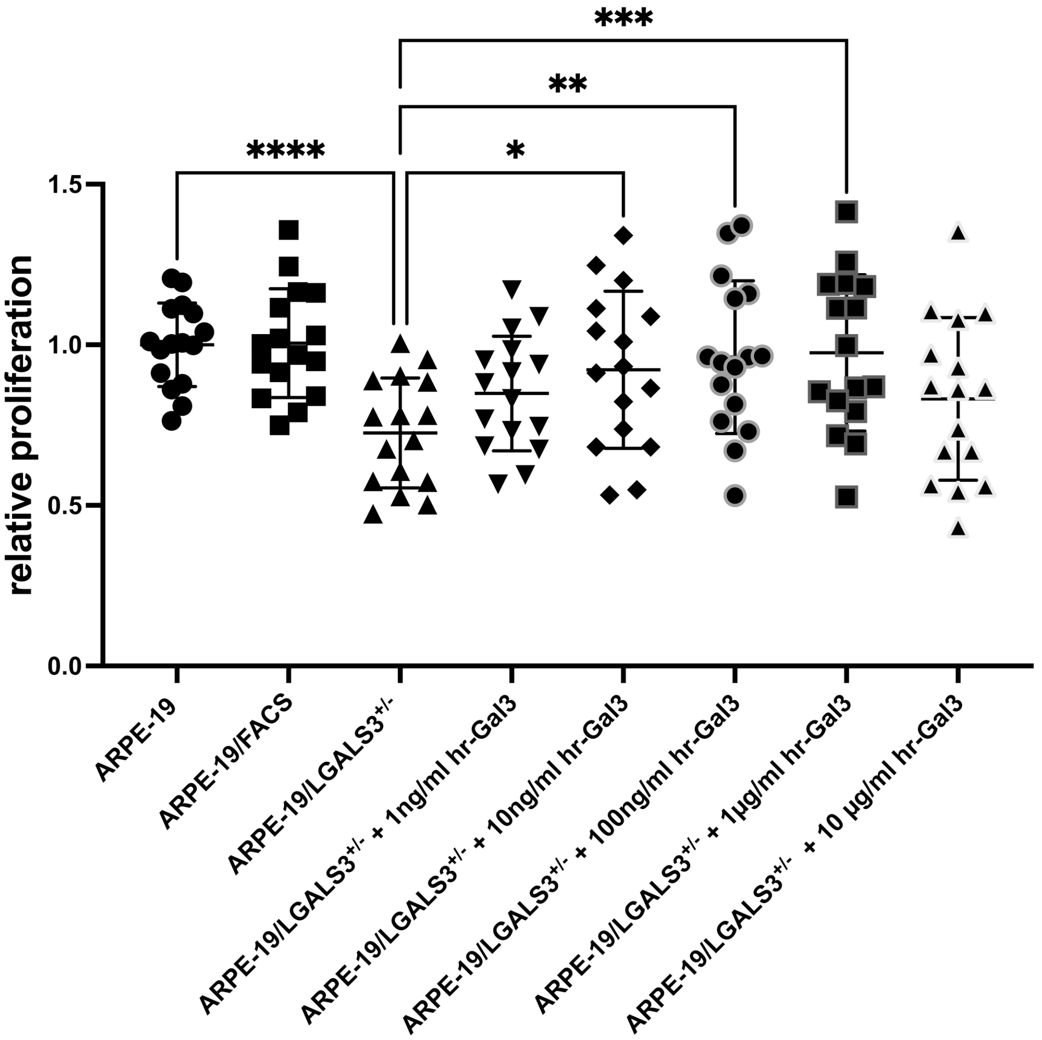

Supplementary Figure S1. Decreased expression of galectin-3 declines proliferation of immortalized RPE cells in vitro. BrdU ELISA of ARPE-19, ARPE-19/FACS and ARPE-19/LGALS3$^{+/-}$ cells following incubation in cell culture medium without supplemented for 72 h with and without various concentration of hr-galectin-3. Mean SD; * p < 0.05; ** p < 0.01; *** p < 0.001; **** p < 0,0001. n = 28 of at least 4 independent experiments.
